# Genotoxicity and glucose tolerance induction by acetyltriethylcitrate, substitute plasticizer compared to di(2-ethylhexyl)phthalate

**DOI:** 10.1038/s41598-019-48599-y

**Published:** 2019-08-22

**Authors:** Jae-Wook Lee, Seok Jong Lee, Myung Chan Gye, Eun-Yi Moon

**Affiliations:** 1 Department of Bioscience and Biotechnology, Sejong University, Seoul, 05006 Republic of Korea; 2 WOOJUNG BIO Co Ltd, Suwon 16229, Gyeonggi-do, Republic of Korea; 3 Department of Life Science and Research Institute of Natural Science, Hanyang University, Seoul, 04763 Republic of Korea

**Keywords:** Environmental monitoring, Risk factors

## Abstract

As di(2-ethylhexyl) phthalate (DEHP), one of phthalates, is classified as probable human carcinogens in EPA, acetyltriethyl citrate(ATEC), one of aliphatic esters, could be applied to DEHP substitute. ATEC is used as plasticizers in cosmetics and nail products. Here, we studied whether ATEC might have genotoxic potential and induce glucose tolerance as compared to DEHP. Genotoxicity was determined by Ames test with histidine-requiring *Salmonella typhimurium* (TA98, TA100, TA1535 and TA1537) and tryptophan-requiring *Escherichia coli* (WP2*uvrA*(pKM101)) strains, chromosomal aberration assay with Chinese hamster lung(CHL/IU) cells, and micronucleus test with bone marrow cells of CD-1 mice. The number of revertants was not significantly changed in Ames test. The frequency of cells with chromosome aberrations was less than 5% in ATEC- or DEHP-treated cells for 6 or 24 h. In addition, no statistically significant increase was observed for the incidence of micronucleated polychromatic erythrocytes (MNPCE) in polychromatic erythrocytes (PCE) and for the ratio of PCE among total erythrocytes at 24 or 48 h after the treatment of mice with ATEC or DEHP. In the meanwhile, blood glucose level (BGL) was increased by the treatment of mice with DEHP or ATEC for 5 consecutive days. Additional 7 days later, BGL by DEHP was recovered to normal level, but not that by ATEC. Then, taken together, our results suggest that ATEC could disrupt glucose metabolism under our experimental conditions. Therefore, although DEHP and ATEC may not be genotoxic, our data should be helpful for persons with the problem in glucose metabolism to choose products containing DEHP or ATEC.

## Introduction

Phthalates are well-known endocrine disrupting chemicals (EDC), which is widely used as effective synthetic plasticizers. DEHP is the most abundant phthalate in a variety of consumer products^1,2^. Phthalates are used as solvents in many applications and in cosmetics^2–4^. However, phthalates have been identified as reproductive and developmental toxicants^1,2^. The most commonly used phthalate is di-(2-ethylhexyl) phthalate (DEHP) in the production of many various manufactures. DEHP is included in medical devices, food wrap, building materials, children’s toys, childcare articles made of polyvinyl chloride (PVC) and cosmetics. DEHP plays a role in holding fragrance, reducing cracking of nail polish, and making products more effectively penetrate and moisturize the skin^1,2,4^. DEHP can migrate into the environment during their production and their use and after disposal^5^. DEHP is one of xenoestrogensns with toxic effects such as reproductive, developmental, and carcinogenic toxicity on both animal and human health^4,6–9^. It has been called those xenoestrogens as an endocrine disruptor (ED). DEHP may induce hepatotoxicity^10,11^ and also enhance tumorigenesis in liver or 1,2-dimethylhydrazine (DMH)-treated colon^12^. Then, US EPA classifies DEHP as probable human carcinogens^13^. In the meanwhile, DEHP exposure induces glucose metabolic disorders^14,15^ and impairs insulin receptor and glucose transporter 4 gene expression^16^. With regard to the reports as above, many efforts are applied to overcome the weakness of DEHP by developing much safer substitute plasticizers.

Triesters of citric acid are considered to be very safe and biocompatible substitutes. Among them, acetyltriethyl citrate (ATEC) functions as plasticizers in cosmetics, mostly in nail products at concentrations up to 7%^17,18^. The results by toxicity studies with ATEC are as follows^17,18^. LD_50_ for ATEC is about 7 ml/kg by oral gavage or 1,150 ± 185 mg/kg by intraperitoneal (IP) administration. No acute oral toxic effect was observed in neuromuscular transmission, body weight, hematological counts, and electrocardiograms. Acute oral toxicity by ATEC was a progressive decrease in blood pressure and heart rate. Intravenous administration of ATEC to cats and rabbits also caused a dose-related loss of blood pressure. No short-term hematological toxicity was caused by IP injection for 14 days with ATEC (230 mg/kg/day). ATEC did not induce skin irritation when inuncted (1 ml/kg body weight) onto intact abdominal rabbit skin daily for 4 days or for 6 days per week for 3 weeks. Minor to moderate changes by ATEC was caused in the eyes for 24 h after its instillation, which had been cleared by 48 h post instillation. ATEC in 3% acacia has resulted in complete, reversible inhibition of sciatic nerve and temporarily abolished corneal reflex action in rabbit eye. ATEC also strongly sensitized guinea-pigs. ATEC induced a low level of cytotoxicity in human HeLa cervical cancer cells. However, no significant changes in lymphoma induction were observed in rats that were fed with ATEC over 2 years^19^.

ATEC brought negative results in Ames test using *Salmonella (S*.*)*. *typhimurium* strains incubated without metabolic activation. In addition, it was non-mutagenic by the assay using L5178Y mouse lymphoma cells in the presence or absence of metabolic activation. No chromosome breakage by ATEC was observed in an *in vivo* cytogenetic assay with cellular suspensions prepared from CD-1 mice. No statistically significant chromosome breakage by ATEC was indicated in an *in vitro* cytogenetic assay performed with cultured human lymphocytes. From all these available data, ATEC was considered safe to be used in cosmetics without genotoxicity in bacterial or mammalian test systems^19,20^. However, little information has been reported about the effect of ATEC on genotoxicity including *in vivo* micronucleus formation compared to DEHP.

Furthermore, genotoxicity that is induced by exposure to toxicants such as cigarette smoke exposure or polyphenols from *Quercus sideroxyla* bark, is associated with metabolic disorders^21,22^. However, no data have been reported about metabolic alteration such as glucose intolerance by ATEC. Therefore, although ATEC did not induce tumor formation and genotoxicity in bacterial and cellular system as above, it is required to clarify whether ATEC could be a substitute plasticizer to DEHP in the face of metabolic changes.

In this study, we thus investigated whether ATEC could induce micronucleus in polychromatic erythrocytes (PCE) as well as bacterial revertants and chromosomal aberration (CA). We used mutant strains, TA98, TA100, TA1535, and TA1537 of *S*. *typhimurium* and *WP2uvrA* (pKM101) of *Escherichia coli*, Chinese hamster lung (CHL/IU) cells and CD-1 mice to assess *in vitro* and *in vivo* genotoxicity. We also investigated glucose tolerance induction by ATEC or DEHP using C57BL/6 mice to measure blood glucose level (BGL).

## Materials and Methods

### Mice and reagents

Specific pathogen-free (SPF) seven weeks old male CD-1 or C57BL6 mice were obtained from ORIENTBIO INC. (Sungnam, Republic of Korea) or DAEHAN Biolink (Cheongjoo, Republic of Korea), respectively. All animals were acclimated for 7 days and observed daily for general health. Five mice were housed in the transparent acrylic cage and maintained in the pathogen-free authorized facility in WOOJUNG BIO CROWISE or in Sejong University where the temperature was at 20–22 °C, the humidity at 50–60%, and a dark/light cycle at 12 h. Mouse-used all experiments were carried out in strict accordance with the guidelines by the recommendations in the Guide for the Care and Use of Laboratory Animals of ‘Animal and Plant Quarantine Agency’, Republic of Korea. The protocol was approved by the Institutional Animal Care and Use Committee, WOOJUNG BIO CROWISE (Permit Number: G31701 for ATEC and G31702 for DEHP) or Sejong University (Permit Number: SJ20160702). All efforts were made to minimize suffering animals.

DEHP, dimethyl sulfoxide (DMSO), sodium azide (SA), 2-nitrofluorene (2-NF), 2-aminoanthracene (2-AA), 9-aminoacridine (9-AA), D-glucose, NaCl, histidine, D-biotin, L-tryptophan, Giemsa solution and sodium carboxymethylcellulose (CMC) was purchased from the Sigma-Aldrich (St. Louis, MO, USA). 2-(2-furyl)-3-(5-nitro-2-furyl) acrylamide(AF2) was purchased from FUJIFILM Wako Chemicals (Osaka, Japan). Acetyltriethylcitrate (ATEC) was purchased from Santa Cruz Biotechnology Inc, (Dallas, TX, USA). Aroclor 1254, mitomycin C (MMC), cyclophosphamide (CP) and colcemid were obtained from Invitrogen (Calsbad, CA, USA). Nutrient broth No.2 was purchased from Oxoid Ltd (Hampshire, UK) and bacto agar was obtained from BD bioscience (San Jose, CA, USA). Except where indicated, all other materials are obtained from Sigma-Aldrich (St. Louis, MO, USA).

### Cell cultures

Chinese hamster lung (CHL/IU) cell (ATCC, CRL-1935) line was obtained from American Type Culture Collection (ATCC, U.S.A.). Cells were maintained in Eagle’s Minimum Essential Medium (EMEM, Lonza Walkersville Inc., U.S.A.) supplemented with 10% heat-inactivated fetal bovine serum (FBS, Invitrogen, U.S.A.), 100 units/ml penicillin and 100 μg/ml streptomycin (Invitrogen, U.S.A.)^4^. Then, cells were incubated to 70–80% confluent at 37 °C with 5% CO_2_ prior to subculture. Mycoplasma contamination was regularly evaluated by Hoechst Stain Kit (MPBIOMEDICALS, Japan). Cells within 13 passages were routinely used to detect chromosomal aberration.

### Bacterial culture

Histidine-requiring *Salmonella typhimurium* (TA98, TA100, TA1535 and TA1537) and tryptophan-requiring *Escherichia coli* (WP2*uvrA*(pKM101)) strains were purchased from Molecular Toxicology, Inc. (MOLTOX^TM^, Boone, NC, USA). Each strain was inoculated into 2.5% nutrient broth No.2 medium and incubated with 90 rpm at 37 °C for 9 h in a shaking incubator. Cultures with a density greater than 1 × 10^9^ cells/ml were used in Ames test.

### Preparation of the test substance

ATEC or DEHP were dissolved in DMSO immediately prior to use. Lower concentrations of ATEC or DEHP were prepared by serial dilution from the highest concentration. The positive control, SA and MMC were dissolved in water or saline. 2-NF, 2-AA, 9-AA, AF2 and CP were dissolved in DMSO. All stock solutions are stored in a deep freezer, below −60 °C, and thawed just prior to use.

### Dose range finding study

For Ames test, a dose range finding study was conducted to establish the highest dose. With the highest dose 5 µl/plate of ATEC and DEHP, following doses were prepared by sequential 2-fold dilution to produce lower dose levels (2.5, 1.25, 0.625, and 0.3125 µl/plate). The highest dose level for main study was justified by the determination of no growth inhibition by ATEC or DEHP in all bacterial strains in the absence and presence of S9 metabolic activation.

### Measurement of cell growth inhibition

For CA assay, cytotoxicity by cell growth inhibition was measured using CHL/IU cells as follows^23^. The highest dose of ATEC or DEHP was 2.0 μl/ml. Sequential dilution was performed to produce additional lower dose levels (1.0, 0.25, 0.0625, and 0.015625 μl/ml). DMSO was used as a negative control. Briefly, cells were resuspended in EMEM and placed with a concentration of 10,000 cells/200 μl/well of 96-well plate (Nunc, Denmark) in a 5% CO_2_ incubator at 37 °C overnight. Then, cells were incubated with 2, 1, 0.25, 0.0625, 0.015625 μl/ml ATEC or DEHP by 6 h- or 24 h-treatment in the absence or presence of S9 metabolic activation mixtures. One group was treated with ATEC or DEHP with or without S9 mix for 6 h, respectively and each well was washed with Dulbecco’s phosphate-buffered saline (D-PBS). Then, a fresh EMEM medium was added and cultured for an additional 18 h. The other group was treated with ATEC or DEHP without S9 mix for 24 h. Assay was performed in quadruplicate for each concentration of ATEC or DEHP. Then, cells were detached with 0.25% Trypsin-EDTA and collected by centrifugation at 150 × g for 5 min. Cell pellets were resuspended and mixed with trypan blue. Total cell number remained in each group was determined with hemocytometer. Cell viability and the value of relative increase in cell counts (RICC) were calculated as follows.

$${\rm{Cell}}\,{\rm{viability}}\,( \% )=\frac{\begin{array}{c}{\rm{Total}}\,{\rm{cell}}\,{\rm{number}}\,{\rm{in}}\,\exp {\rm{.}}\,{\rm{group}}\end{array}}{{\rm{Total}}\,{\rm{cell}}\,{\rm{number}}\,{\rm{in}}\,{\rm{control}}\,{\rm{group}}}\times 100$$

$${\rm{RICC}}\,( \% )=\frac{{\rm{Increased}}\,{\rm{cell}}\,{\rm{number}}\,{\rm{in}}\,\exp {\rm{.}}\,{\rm{group}}}{{\rm{Increased}}\,{\rm{cell}}\,{\rm{number}}\,{\rm{in}}\,{\rm{control}}\,{\rm{group}}}\times 100$$

In addition, relative population doubling (RPD) in experimental group was calculated from population doubling (PD) in control group as below. PD is the log of the ratio of the final cell count to the starting (initial baseline) cell count, divided by the log of 2; that is PD = [log(Cell count_final_/Cell count_initial_)]/log 2^24,25^.

$${\rm{RPD}}\,( \% )=\frac{{\rm{PD}}\,{\rm{in}}\,\exp {\rm{.}}\,{\rm{group}}}{{\rm{PD}}\,{\rm{in}}\,{\rm{control}}\,{\rm{group}}}\times 100$$

### Preparation of minimal glucose agar plate and top agar

Minimum glucose agar plates were prepared from mixture of the autoclaved Bacto agar (15 g in Ultra pure water 930 ml), 50 ml 40% D-(+)-glucose and 20 ml sterile 50X Vogel-Bonner salts (MgSO_4_·7H_2_O 1 g, Citric acid 10 g, K_2_HPO_4_ 50 g NaNH_5_PO_4_·4H_2_O 17.5 g in Ultra pure water 100 ml). Top agar that contained 0.6% Bacto agar and 0.5% NaCl was autoclaved and mixed with 0.5 mM L-Histidine/D-Biotin (Sigma-Aldrich, U.S.A.) at a ratio of 10 to 1 for *Salmonella typhimurium* and with 0.5 mM L-Tryptophan (Sigma-Aldrich, U.S.A.) solution at a ratio of 10 to 1 for *Escherichia coli*, respectively.

### Preparation of S9 mix

Mutazyme S9 mix including NADPH cofactors was purchased from Molecular Toxicology, Inc. (MOLTOX^TM^, Boone, NC, USA) and stored below −20 °C until use. S9 mix was prepared from Sprague-Dawley rat liver induced with Aroclor 1254. For Ames test, 500 μL 5% S9 mix was used to mix 100 μl of each test substance solution, 100 μl of each bacterial suspension and 2 ml top agar. For chromosomal aberration (CA) assay, the final concentration of S9 mix was 1% for 6 h-treatment.

### Ames test

Ames test was performed by using histidine-requiring *Salmonella typhimurium* (TA98, TA100, TA1535 and TA1537) and tryptophan-requiring *Escherichia coli* (WP2*uvrA*(pKM101)) strains as follows^26–28^. In the presence of S9 metabolic activation, 100 μl of each test substance solution, the negative control and strain-specific positive control were placed in glass tubes sterilized by dry oven. 500 μl S9 mix and 100 μl of each bacterial suspension were mixed and incubated in a shaking water bath at 37 °C for 20 min. Then, 2 ml top agar containing each bacterial strain was added and mixed thoroughly with a vortex mixer. Lastly, this mixture was poured into minimal glucose agar plate and allowed to solidify at room temperature. In absence metabolic activation, experimental method was identical to above except the use of 500 μl of 0.1 M phosphate buffer (pH 7.4) instead of S9 mix. After solidification of the top agar, the minimal glucose agar plate was incubated by upside down at 37 °C for 48 h.

When the number of revertant colonies was counted manually, each experiment should be validated by at least twice higher number of revertant colonies in positive control group without (Table 1) or with (Table 2) S9 than those in negative control group. In addition, at least 4 dose levels did not exhibit growth inhibition and all plates did not show any evidence of contamination. Then, when the number of revertant colonies in any strains at one or more doses is at least twice higher than that in negative control, the results in experimental groups were considered to be positive. It should be also increased as dose dependency or reproducibility.

**Table 1:** Number of revertant colonies for each bacterial strain by positive control without S9 mix.

Bacterial strain		Positive control	S9 mix	Concentration (μg/plate)	Number of revertants
*Salmonella* *typhimurium*	TA98	2-Nitrofluorene (2-NF)	−	0	19 ± 5.6
				1	170.3 ± 17.5*
	TA100	Sodium azide (SA)	−	0	93.3 ± 2.9
				0.5	474.7 ± 26.1*
	TA1535	Sodium azide (SA)	−	0	7.3 ± 2.1
				0.5	96.0 ± 4.4*
	TA1537	9-Aminoacridine (9-AA)	−	0	4.3 ± 2.5
				80	520.0 ± 55.7*
*Escherichia coli*	WP2*uvr*A	2-(2-furyl)-3-(5-nitro-2-furyl) acrylamide (AF2)	−	0	142.0 ± 4.4
				0.005	1,316 ± 46.1*

Bacterial revertant formation was measured in triplicate by Ames test. *Salmonella typhimurium* (TA98, TA100, TA1535 and TA1537) were treated with 2-NF, SA, and 9-AA in the absence of S9 mix. *Escherichia coli* (WP2*uvrA*(pKM101)) strains were treated with AF2 in the absence of S9 mix. The number of revertant colonies was counted manually. *When the result in experimental group was at least twice higher than that in negative control, it was considered to be positive.

**Table 2:** Number of revertant colonies for each bacterial strain by positive control with S9 mix.

Bacterial strain		Positive control	S9 mix	Concentration (μg/plate)	Number of revertants
*Salmonella* *typhimurium*	TA98	2-Aminoanthracene (2-AA)	+	0	17.5 ± 2.1
				0.5	100.0 ± 11.3*
	TA100	2-Aminoanthracene (2-AA)	+	0	115.0 ± 5.7
				1	605.0 ± 9.9*
	TA1535	2-Aminoanthracene (2-AA)	+	0	9.0 ± 1.4
				2	55.0 ± 5.7*
	TA1537	2-Aminoanthracene (2-AA)	+	0	3.0 ± 1.4
				2	77.5 ± 0.7*
*Escherichia coli*	WP2*uvr*A	2-Aminoanthracene (2-AA)	+	0	110.5 ± 2.1
				2	784.0 ± 101.8*

Bacterial revertant formation was measured in triplicate by Ames test. *Salmonella typhimurium* (TA98, TA100, TA1535 and TA1537) and *Escherichia coli* (WP2*uvrA*(pKM101)) strains were treated with 2-AA in the presence of S9 mix. The number of revertant colonies was counted manually. *When the result in experimental group was at least twice higher than that in negative control, it was considered to be positive.

### Chromosomal aberration assay

Chromosomal aberration was assessed as follows^29^. The highest dose level for main study was determined from the value of RICC, which is calculated at the section of ‘Measurement of cell growth inhibition’. Additional lower dose levels were prepared by 2-fold serial dilution (Table 3). Briefly, CHL/IU cells were placed at 2.5 × 10^5^ cells/5 ml in a 60 mm^2^ plate (BD, USA.) and incubated in a 5% CO_2_ incubator at 37 °C overnight. One group was treated with ATEC or DEHP with or without S9 mix for 6 h, respectively and each well was washed with D-PBS. Then, a fresh EMEM medium was added and cultured for an additional 18 h. The other group was treated with ATEC or DEHP without S9 mix for 24 h. Assay was performed in quadruplicate for each concentration of ATEC or DEHP. Cells were arrested in metaphase by the addition of 0.2 μg/ml of colcemid (Invitrogen, U.S.A.) at 2 h before cell harvest. Cells were collected by detachment with 0.25% trypsin-EDTA and by centrifugation at 150 × g for 5 min. Then, cells were incubated in 0.075 M KCl hypotonic solution at 37 °C for 20 min and fixed with ice-cold fixative (methanol : acetic acid, 3 : 1). One or two drops of the suspension were placed on slide glass. Cells in each slide were air-dried and stained with 3% Giemsa solution in 0.01 M Sörenson phosphate buffer (pH 6.8) for 20 min.

**Table 3:** Doses to assess chromosomal aberration.

Group	S9 mix	Doses for main experiment (μl/ml)	
		ATEC	DEHP
6 h-treatment	−	0.7, 0.35, 0.175, 0.13	0.13, 0.065, 0.0325
	+	1.3, 1, 0.65, 0.325	2, 1, 0.5
24 h-treatment	−	0.2, 0.1, 0.05, 0.0156	0.0156, 0.0078, 0.0039

Main experiment for chromosomal aberration was performed with various doses of ATEC and DEHP. CHL cells were treated with ATEC or DEHP for 6 or 24 h in the presence or absence of S9 mix.

Chromosomes in 200 metaphases were evaluated for each concentration as follows; Each cells in metaphase was observed under inverted microscope (BX53, Olympus, Japan) at a magnification of 400x or 1,000x. Any cell with one or more structural and numerical aberrations was counted as one aberrant cell. Structural chromosomal aberrations were classified into chromatid break (ctb), chromatid exchange (cte), chromosome break (csb), chromosome exchange (cse), chromatid or chromosome gap (gap). When several gaps or breaks were evident in metaphase, these were recorded as a fragment (frg). Gaps (g) were not be recorded as structural aberrations and were not included in the calculation of the aberration rates. An achromatic lesion narrower than the width of one chromatid was recorded as a gap. The frequency of numerical aberrations (polyploid; pol, endoreduplication; end) were recorded.

The frequency of cells with chromosome aberration except gaps was determined in accordance with the criteria of Toshio Sofuni^30,31^. The frequency of cells with <5% chromosome aberrations was negative, >10%, positive and 5~10%, equivocal positive (±). In addition, the dose levels which had more than 200 metaphases should be above three and the cultures did not show any evidence of contamination.

### Micronucleus test

Mice were orally administered with 2,000, 1,000, 500 mg/kg of ATEC or DEHP suspended in 0.5% carboxymethylcellulose (CMC) solution. Mice in positive control group were intraperitonelly injected with 2 mg/kg MMC dissolved in saline solution. Body weight, clinical signs and mortality were recorded immediately at 2 h, day 1, 2 and 3 after the injection of each material. All animals were sacrificed by cervical dislocation and bone marrow cells were collected by rinsing femur canal with 200 µl FBS at 24 h after the injection of test materials. Cells were centrifuged at 150 xg for 5 min. and cell pellets were dispersed well. One drop of the suspensions was placed on clean dry slides and spread. The slides were air-dried, fixed with methanol for 5 min. and stained with a 3% Giemsa staining solution in 0.01 M Sörenson phosphate buffer solutions (pH 6.8) for 30 min. The stained slides were washed with 0.01 M Sörenson phosphate buffer solution (pH 6.8) and 0.004% of citric acid solution. Then, the slides were air-dried.

Polychromatic erythrocytes (PCE) were observed under a fluorescence microscope (BX51, Olympus, Japan) at a magnification of 1,000x. The number of micronucleated polychromatic erythrocyte (MNPCE) in 2,000 PCE was recorded. Index of bone marrow cytotoxicity was calculated by the ratio of PCE to the total number of erythrocytes. Data were validated by the evaluation that the number of MNPCE in 2,000 PCE in positive control group was statistically increased as compared to that in negative control group^32,33^.

### Glucose tolerance test

Changes in blood glucose level (BGL) were measured by glucose tolerance test (GTT) as follows. At least 4 mice were housed for each experiment group. Mice in each group were administered with 4, 40, 400, 2,000 mg/kg ATEC or DEHP by oral gavage for 5 consecutive days. On one day before GTT experiment, mice were moved into fresh cage and fasted only with water supply for 18 h after the last administration. BGL was measured by using Accu-check active glucometer (Roche, Basel, Switzerland) after blood was collected from each mouse tail vein. 20% D-glucose solution was prepared and sterilized by using 0.2 µm filter. Then, mice were injected intraperitoneally with 10 µl of 20% D-glucose solution for 1 g of body weight at 18 h after the fasting. BGL was measured at 15, 30, 60, 90 and 120 min after glucose injection. GTT should include the basal level of BGL without injecting 20% D-glucose solution before main experiment. Changes in BGL were represented with line graph.

### Statistical analyses

For Ames test, statistical analysis was not performed but individual plate counts, averages and standard deviations of revertant colonies are presented. For CA assay, Fisher’s exact method was used for comparison of the negative control group with the experimental or the positive control groups^30,31,34^. For micronucleus test, the criteria of Kastenbaum and Bowman was used to analyze the significant positive increase in the number of MNPCE^33^. Homogeneity of variance in the frequency of PCE and body weight was analyzed by using Bartlett’s test. In addition, one-way analysis of variance (ANOVA) was employed for homogeneous data; then, if significant, Dunnett’s t-test was applied for multiple comparisons. *P* value of <0.05 or <0.01 was considered to be significant.

## Results

### ATEC and DEHP did not induce the formation of revertant colonies

**Figure 1:**
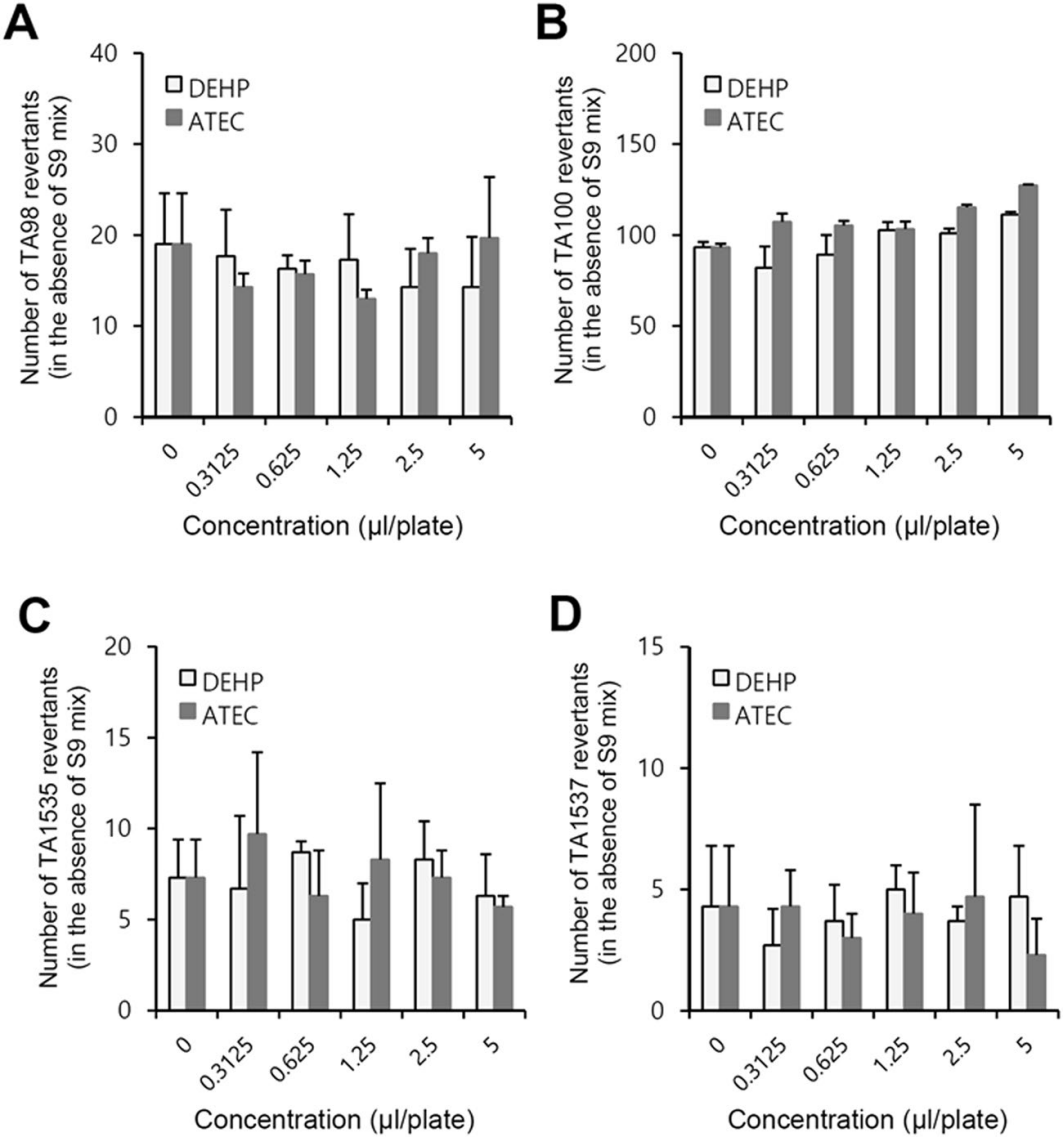
Dose range finding study was conducted to define dose levels of ATEC or DEHP for the test with *Salmonella typhimurium*. (**A**–**D**) Bacterial revertant formation was measured in triplicate by Ames test. *Salmonella typhimurium* TA98 (**A**), TA100 (**B**), TA1535 (**C**) and TA1537 (**D**) strains were treated with ATEC or DEHP in the absence of S9 mix. The number of revertant colonies was counted manually. Data in bar graph represent mean ± SED (n = 3). When the results in experimental groups were at least twice higher than that in negative control, it was considered to be positive.

**Figure 2:**
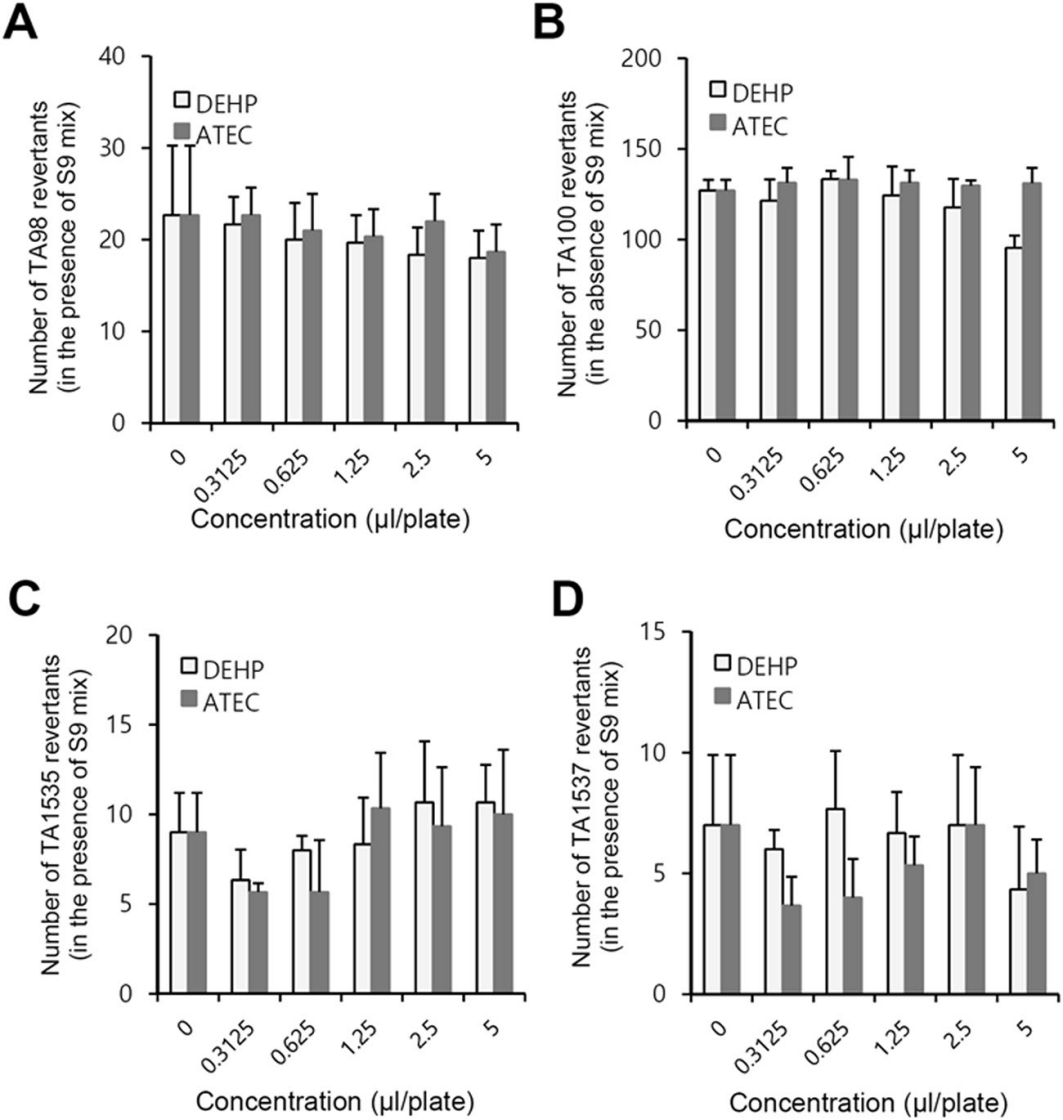
Main study was conducted to measure the number of revertants for *Salmonella typhimurium* treated with ATEC or DEHP. (**A**–**D**) *Salmonella typhimurium* TA98 (**A**), TA100 (**B**), TA1535 (**C**) and TA1537 (**D**) strains were treated with ATEC or DEHP in the presence of S9 mix. The number of revertant colonies was counted manually. Data in bar graph represent mean ± SED (n = 3). When the result in experimental group was at least twice higher than that in negative control, it was considered to be positive.

**Figure 3:**
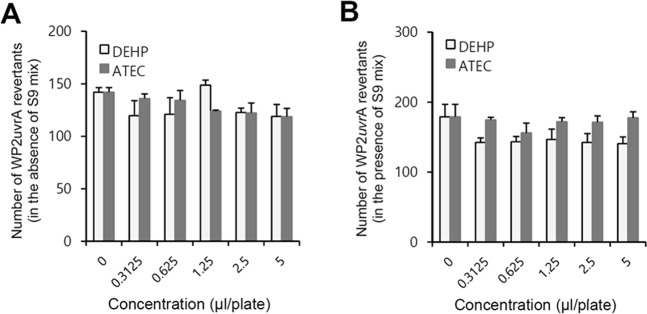
Bacterial revertants of *Escherichia coli* were measured by the treatment with ATEC or DEHP in the absence or presence of metabolic activation. (**A**,**B**) Bacterial revertant formation was measured in triplicate by Ames test. *Escherichia coli* (WP2*uvrA*(pKM101)) strains were treated with ATEC or DEHP without (**A**) or with (**B**) S9 mix. The number of revertant colonies was counted manually. Data in bar graph represent mean ± SED (n = 3). When the result was at least twice higher than that in negative control, it was considered to be positive.

To determine the mutagenic potential of ATEC and DEHP, the bacterial revertant formation was measured in triplicate by Ames test. First of all, the dose range finding study was conducted to define dose levels of the main study using *Salmonella typhimurium* (TA98, TA100, TA1535 and TA1537) or *Escherichia coli* (WP2*uvrA* (pKM101)) and various doses (5, 2.5, 1.25, 0.625, and 0.3125 μl/plate) selected for ATEC and DEHP in the absence and presence of metabolic activation with S9 mix. No changes in bacterial growth were detected by ATEC or DEHP at all dose levels (data not shown). In addition, no contaminations were observed in all bacterial cultures (data not shown). Then, the main study of Ames test using *Salmonella typhimurium* was performed with all doses of ATEC or DEHP in the absence (Fig. 1) or presence (Fig. 2) of S9 mix. The increase in bacterial revertants of *Salmonella typhimurium* was not observed by the treatment with ATEC in the absence of metabolic activation compared to DEHP. As shown in Fig. 1, the mean number of revertant colonies was not more than twice in the group of ATEC- or DEHP-treated groups as compared to those in negative control group. In addition, no increase in bacterial revertants of *Salmonella typhimurium* was observed by the treatment with ATEC in the presence of metabolic activation compared to DEHP (Fig. 2). The mean number of revertant colonies was not more than twice in the group of ATEC- or DEHP-treated groups as compared to those in negative control group. When Ames test using *Escherichia coli* (WP2*uvrA* (pKM101)) was also performed with all doses of ATEC or DEHP in the absence or presence of S9 mix, bacterial revertants of *Escherichia coli* was not increased by the treatment with ATEC in the absence or presence of metabolic activation. The mean number of revertant colonies was not more than twice in the group of ATEC- or DEHP-treated groups as compared to those in negative control group (Fig. 3). Concurrently, when positive and negative control groups were also tested in the absence (Table 1) or presence (Table 2) of S9 mix, the mean number of revertant colonies in positive control group was markedly increased as compared to those in negative control group. The results suggest that ATEC or DEHP did not exhibit the indications of mutagenic potential under our experimental conditions. It suggests that genotoxic effect of ATEC is comparable to DEHP.

### ATEC and DEHP did not trigger chromosomal aberration

**Figure 4:**
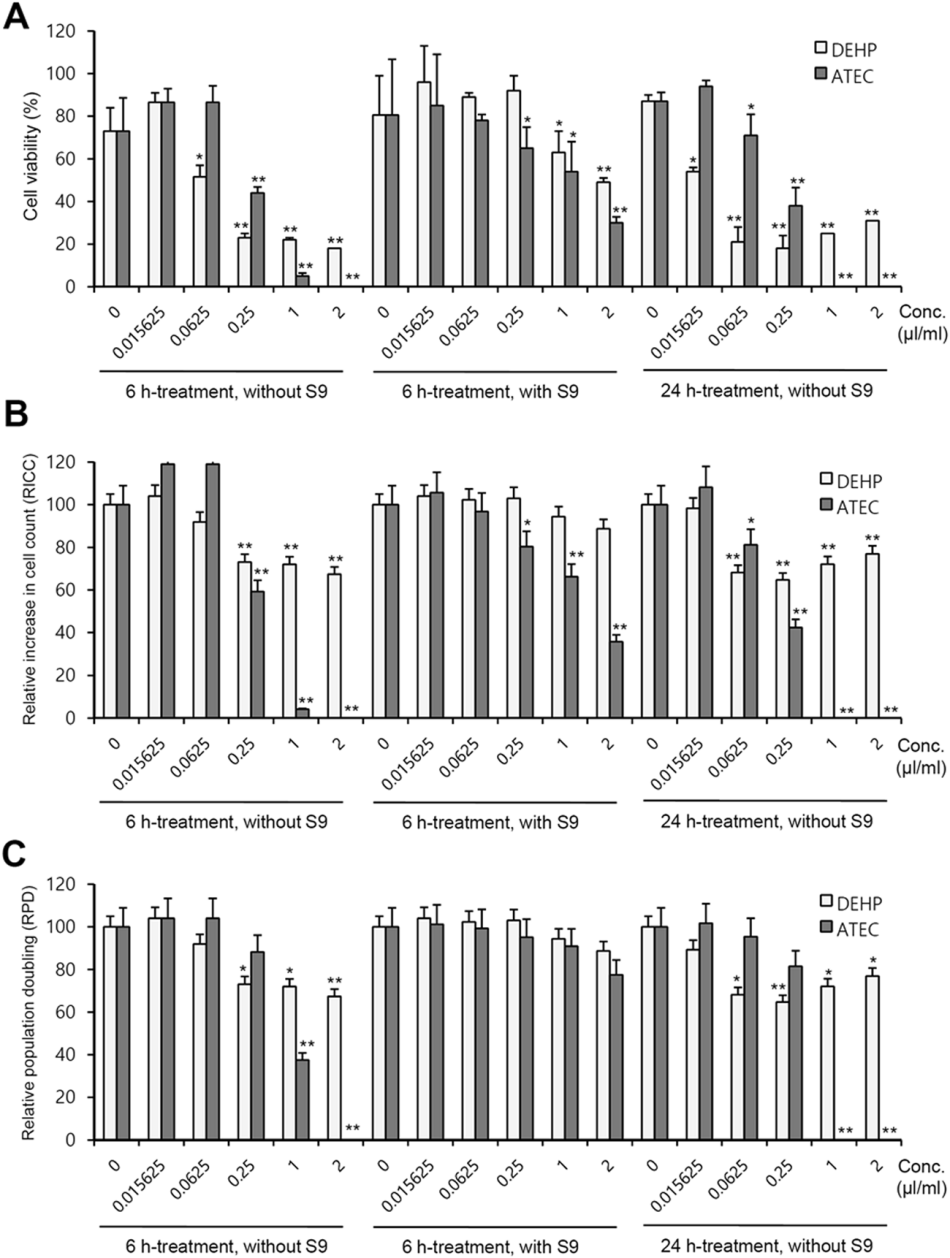
Cytotoxic effect of ATEC or DEHP was measured in Chinese hamster lung (CHL/IU) cells. (**A**–**C**) CHL/IU cells were treated with ATEC or DEHP without or with S9 mix for 6 h, respectively. Each well was washed with Dulbecco’s phosphate-buffered saline (D-PBS). Then, a fresh EMEM medium was added and cultured for an additional 18 h. The other group was treated with ATEC or DEHP without S9 mix for 24 h. For each group, cell suspension was mixed with trypan blue and total cell number was counted with hemocytometer. Cell viability (**A**), RICC (**B**) and RPD (**C**) were calculated by the methods described in materials and methods. Data in bar graph represent mean ± SED (n = 4). *p < 0.05; **p < 0.01, significant difference as compared to DEHP- or ATEC-untreated control group at each experimental condition.

We examined the cytotoxicity of ATEC or DEHP on CHL/IU cells by the incubation with various those concentrations for 6 or 24 h in the absence or presence of S9 mixtures (mix). Then, the percentage of cell viability, RPD and RICC were calculated and the dose levels for main study were determined from RICC that is not showing growth inhibition. DEHP was cytotoxic over 1.0 μl/ml in 6 h-treated cells with S9 mix (Fig. 4A, middle). and 0.0625 or 0.015625 μl/ml in 6 h- or 24 h-treated group without S9 mix, respectively. ATEC was cytotoxic over 0.25 μl/ml in 6 h-treated cells with S9 mix and 0.25 or 0.0625 μl/ml in 6 h- or 24 h-treated group without S9 mix, respectively (Fig. 4A, left and right). No RICC changes in DEHP- or ATEC-treated group were respectively observed below 2.0 or 0.0625 μl/ml in 6 h-treated cells with S9 (Fig. 4B, middle). No RICC changes in both DEHP- and ATEC-treated group were observed below 0.0625 or 0.015625 μl/ml in 6 h- or 24 h-treated cells without S9 mix, respectively (Fig. 4B, left and right). No RPD changes in both DEHP- and ATEC-treated group were observed below 2.0 μl/ml in 6 h-treated cells with S9 (Fig. 4C, middle). No RPD changes in DEHP-treated group were observed below 0.0625 or 0.015625 μl/ml in 6 h- or 24 h-treated cells without S9 mix, respectively. No RPD changes in ATEC-treated group were observed below 0.25 μl/ml in both 6 h- and 24 h-treated cells without S9 mix (Fig. 4C, left and right). Data suggest that ATEC could be less cycotoxic for CHL/IU cells compared to DEHP.

Based on the results of the growth inhibition, the highest dose levels for ATEC were calculated from its RICC. The highest doses were 0.7 μg/ml for 6 h-treatment with S9 mix, 1.2 or 0.2 μg/ml for 6 h- or 24 h-treatment without S9 mix, respectively. The highest dose levels of DEHP were 0.13, 2.0 and 0.0156 μg/ml for each same condition like in ATEC-treated group (Table 3). Then, chromosomal aberration was measured with the highest dose and three additional lower doses that were prepared by 2-fold serial dilution. The frequency of cells with chromosome aberrations was less than 5% for each experimental group. No significant differences were observed in the frequency of cells with chromosome aberrations in any dose levels of ATEC (Table 4) or DEHP (Table 5) compared to that in negative control group. In contrast, positive control groups with 10 μg/ml CP or 0.05 μg/ml MMC showed the significant increase in the frequency of cells with structural chromosomal aberrations compared to that in negative control group (Tables 4 and 5). Data suggest that ATEC may not alter chromosome structure, which is comparable to DEHP.

**Table 4:** Chromosomal aberrations by ATEC in Chinese Hamster Lung (CHL/IU) cells.

Sample	Concentration	S9 mix	Treatment time (h)	Number of cells with structural aberrations						gap (%)
				csb	ctb	cse	cte	frg	total (%)	
ATEC (μl/ml)	0	−	6	0	0	0	0	0	0 (0.0)	0 (0.0)
	0.13	−	6	0	1	0	0	0	1 (2.0)	0 (0.0)
	0.175	−	6	0	0	0	0	0	0 (0.0)	0 (0.0)
	0.35	−	6	0	0	0	0	0	0 (0.0)	0 (0.0)
	0.7	−	6	0	0	0	0	1	1 (2.0)	0 (0.0)
MMC (μg/ml)	0.05	−	6	1	4	0	6	0	11* (22.0)	1 (2.0)
ATEC (μl/ml)	0	+	6	0	0	0	0	0	0 (0.0)	0 (0.0)
	0.325	+	6	0	0	0	0	0	0 (0.0)	2 (4.0)
	0.65	+	6	0	0	0	0	0	0 (0.0)	0 (0.0)
	1	+	6	0	0	0	0	0	0 (0.0)	0 (0.0)
	1.3	+	6	0	0	0	0	0	0 (0.0)	0 (0.0)
CP (μg/ml)	10	+	6	2	2	0	6	0	10* (20.0)	0 (0.0)
ATEC (μl/ml)	0	−	24	0	0	0	0	0	0 (0.0)	0 (0.0)
	0.0156	−	24	0	0	0	0	0	0 (0.0)	0 (0.0)
	0.05	−	24	1	0	0	0	0	1 (2.0)	0 (0.0)
	0.1	−	24	0	0	0	0	0	0 (0.0)	0 (0.0)
	0.2	−	24	0	0	0	0	0	0 (0.0)	1 (2.0)
MMC (μg/ml)	0.05	−	24	2	3	0	8	0	13* (26.0)	0 (0.0)

Number of cells with structural aberrations, chromatid break (ctb), chromatid exchange (cte), chromosome break (csb), chromosome exchange (cse), and chromatid or chromosome gap (gap) was counted under inverted microscope. *Total frequency of structural aberrations was significantly increased (>10%) in positive control groups treated with mitomycin C (MMC) or cyclophosphamide (CP).

**Table 5:** Chromosomal aberrations by DEHP in Chinese Hamster Lung (CHL/IU) cells.

Sample	Concentration	S9 mix	Treatment time (h)	Number of cells with structural aberrations						gap (%)
				csb	ctb	cse	cte	frg	total (%)	
DEHP (μl/ml)	0	−	6	0	0	0	0	0	0 (0.0)	0 (0.0)
	0.0325	−	6	0	0	0	0	0	0 (0.0)	0 (0.0)
	0.065	−	6	0	0	0	0	0	0 (0.0)	0 (0.0)
	0.13	−	6	0	0	0	0	0	0 (0.0)	1 (2.0)
MMC (μg/ml)	0.05	−	6	2	7	1	5	0	15* (30.0)	0 (0.0)
DEHP (μl/ml)	0	+	6	0	0	0	0	0	0 (0.0)	0 (0.0)
	0.5	+	6	0	0	0	0	0	0 (0.0)	0 (0.0)
	1	+	6	0	0	0	0	0	0 (0.0)	0 (0.0)
	2	+	6	0	0	0	0	0	0 (0.0)	0 (0.0)
CP (μg/ml)	10	+	6	0	5	0	7	0	12* (24.0)	0 (0.0)
DEHP (μl/ml)	0	−	24	0	0	0	0	0	0 (0.0)	0 (0.0)
	0.0039	−	24	0	0	0	0	0	0 (0.0)	0 (0.0)
	0.0078	−	24	0	1	0	0	0	1 (2.0)	0 (0.0)
	0.0156	−	24	0	0	0	0	0	0 (0.0)	1 (2.0)
MMC (μg/ml)	0.05	−	24	1	5	0	8	0	14* (28.0)	0 (0.0)

Number of cells with structural aberrations, chromatid break (ctb), chromatid exchange (cte), chromosome break (csb), chromosome exchange (cse), and chromatid or chromosome gap (gap) was counted under inverted microscope. *Total frequency of structural aberrations was significantly increased (>10%) in positive control groups treated with mitomycin C (MMC) or cyclophosphamide (CP).

### ATEC and DEHP did not derive micronucleus in polychromatic erythrocytes

We tested mutagenic potential of ATEC and DEHP by micronucleus formation in mouse bone marrow cells. Mice were treated with 2,000 mg/kg by oral gavage that is an intended route of administration in human. No mortality and abnormal clinical signs in each animal were observed at any dose levels (data not shown). Due to no sex differences of micronucleus formation in the preliminary study, female mice were not used in main experiment (data not shown). No significant differences in body weight were determined at 24, 48 and 72 h after the last administration by oral gavage as compared to those in control group (data not shown). No statistically significant increases in the incidence of micronucleated polychromatic erythrocytes (MNPCE) in polychromatic erythrocytes (PCE) were noted compared to control group at each time point (Table 6). Then, we examined dose-dependent micronucleus formation by ATEC and DEHP in mouse bone marrow cells. Mice were treated with 500, 1000 and 2000 mg/kg by oral gavage. While no significant incidence of MNPCE in PCE was observed in groups treated with ATEC or DEHP, it was significantly increased in MMC-treated group compared to control group (Table 7). No statistically significant differences in the ratio of PCE among total erythrocytes were noted at 24, 48 and 72 h after administration of ATEC, DEHP or MMC compared to control group (Table6 and 7). Based on these results, it suggests that ATEC may not have any potential to induce micronuclei formation in PCE from mouse bone marrow under our experimental conditions, which is comparable to the results with DEHP.

**Table 6:** Number of micronucleus in polychromatic erythrocytes, time-dependently.

Group	Sampling time (h)	PCE/(PCE + NCE)	MNPCE/PCE
Vehicle	24	0.324 ± 0.018	0.8 ± 1.3
DEHP (2,000 mg/kg)	24	0.274 ± 0.046	0.0 ± 0.0
	48	0.305 ± 0.022	0.0 ± 0.0
	72	0.288 ± 0.018	0.0 ± 0.0
ATEC (2,000 mg/kg)	24	0.276 ± 0.052	0.3 ± 0.6
	48	0.298 ± 0.023	0.3 ± 0.6
	72	0.242 ± 0.024	0.0 ± 0.0
MMC (2 mg/kg)	24	0.344 ± 0.026	97.6 ± 6.5^†,*^

MNPCE: Micronucleated polychromatic erythrocyte.

PCE: Polychromatic erythrocyte.

NCE: Normochromatic erythrocyte.

MMC: Mitomycin C.

Significant difference from negative control by Kastendaum & Bowman: ^†^p < 0.05.

Significant difference from negative control by Dunnett’s t-test: *p < 0.05.

**Table 7:** Number of micronucleus in polychromatic erythrocytes, dose-dependently.

Group	Dose (mg/kg)	PCE/(PCE + NCE)	MNPCE/PCE
Vehicle	0	0.341 ± 0.019	0.4 ± 0.7
DEHP	500	0.384 ± 0.052	0.4 ± 0.5
	1000	0.381 ± 0.031	0.2 ± 0.4
	2000	0.346 ± 0.029	0.4 ± 0.5
ATEC	500	0.379 ± 0.066	0.4 ± 0.5
	1000	0.372 ± 0.051	1.0 ± 1.0
	2000	0.326 ± 0.031	0.2 ± 0.4
MMC	2	0.348 ± 0.027	100 ± 7.35^†,*^

MNPCE: Micronucleated polychromatic erythrocyte.

PCE: Polychromatic erythrocyte.

NCE: Normochromatic erythrocyte.

MMC: Mitomycin C.

Significant difference from negative control by Kastendaum & Bowman: ^†^p < 0.05.

Significant difference from negative control by Dunnett’s t-test: *p < 0.05.

### Glucose tolerance induction by ATEC was higher than that by DEHP

**Figure 5:**
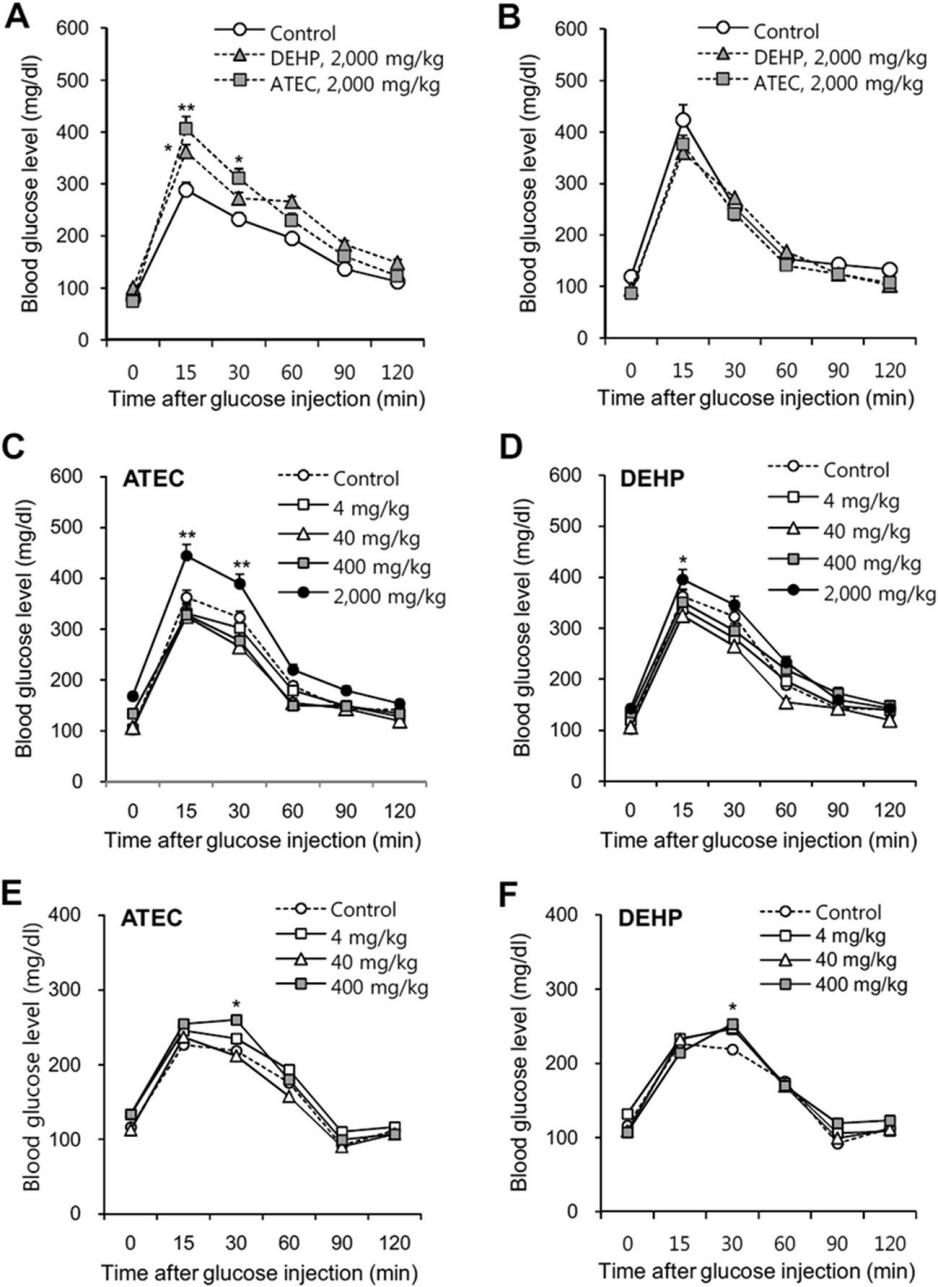
*In vivo* changes in blood glucose level (BGL) were measured by the administration with ATEC or DEHP. (**A**–**F**) Various doses of 2,000 (**A**–**D**), 400, 40 and 4 (**C**–**F**) mg/kg ATEC (**A**–**C**,**E**) or DEHP (**A**,**B**,**D**,**F**) and saline in control group were administered into mice by oral gavage for 5 consecutive days (**A**,**C**,**D**). Experiments in (**C**,**D**) were simultaneously performed with the same saline-administered control group. An additional single dose was applied to the same mice 7 days after the last administration (**B**,**E**,**F**). Experiments in (**E**,**F**) were simultaneously performed with the same saline-administered control group. BGL was measured in blood collected at 15, 30, 60, 90 and 120 min from each mouse which were fasted for 18 h and injected with 20% D-glucose solution. Changes in BGL were represented with line graph. Data in line graph represent mean ± SED (n = 4). *p < 0.05; **p < 0.01, significant difference as compared to control group at each time point.

Due to the results with no genotoxicity, we examined the physiological effect of ATEC and DEHP by the measurement of blood glucose level (BGL). When mice were administered with 2,000 mg/kg ATEC or DEHP by oral gavage for 5 consecutive days, BGL was increased significantly by ATEC, which was higher than that by DEHP (Fig. 5A,5C and 5D). Then, when mice were acclimated without administration of 2,000 mg/kg ATEC or DEHP for 7 days, then injected with the same dose once, changes in BGL was not observed compared to control group (Fig. 5B). In contrast, although no changes in BGL were detected by the administration with 4, 40, and 400 mg/kg of ATEC or DEHP for 5 consecutive days (Fig. 5C and 5D), a significant changes in BGL was observed by an additional single administration of 400 mg/kg ATEC at the 7^th^ day after the last administration but not significant by 4, or 40 mg/kg (Fig. 5E). Little changes in BGL except at 30 min were also detected at the 7th day after the last administration with 4, 40, and 400 mg/kg of DEHP (Fig. 5F). Data demonstrate that the effect of ATEC on BGL was a bit higher than that by DEHP and glucose tolerance induction by ATEC might be associated with period and amount of its exposure. The results also implicate that ATEC, which might differently affect BGL depending on the exposure level and the individual pre-exposed to ATEC could be tolerable to the responses in BGL changes.

## Discussion

Human body could be influenced by EDCs that is contained in many products. Phthalates are well-known EDC, which is widely used as effective synthetic plasticizers. DEHP is the most abundant phthalate in a variety of consumer products^1,2^. DEHP has toxic effects on both animal and human health^4,6–9^. DEHP may enhance tumorigenesis^4,12^ and it is classified as probable human carcinogens in US EPA^13^. DEHP exposure induces glucose metabolic disorders^14,15^ and impairs insulin receptor and glucose transporter 4 gene expression^16^. So, many substitute plasticizers are developed to overcome the weakness of DEHP. ATEC, one of triester of citric acid, is comparably safe and functions as substitute plasticizers in cosmetics^17,18^. Some reports showed that metabolic disorders are associated with genotoxicity by toxicants^21,22^. So, in this study, we investigated whether ATEC could be a better substitute plasticizer than DEHP as judged by *in vivo* genotoxicity. We also determined the metabolic changes by ATEC and their relationship with genotoxicity in comparison with DEHP.

Since our data did not show genotoxicity by ATEC or DEHP *in vitro* and *in vivo*, these indicate non-mutagenic potential of ATEC or DEHP under our experimental conditions. The number of revertant bacterial colonies increased by ATEC was insignificant as compared to those in control group. No significant difference was shown in revertant bacterial colonies by DEHP. In addition, lower cycotoxicity in CHL/IU cells was observed by ATEC as compared to that by DEHP. Chromosome structures may not be altered by ATEC or DEHP. ATEC may not induce micronuclei formation in PCE, which is comparable to the results with DEHP. It suggests that non-genotoxic activity of ATEC is comparable to DEHP.

However, while the low dose of ATEC did not affect BGL, the high dosage of ATEC enhanced BGL. These implicate that glucose tolerance induction by ATEC might be associated with period and amount of its exposure. Then, it suggests that the low amount of ATEC was comparably safe to metabolic changes by glucose tolerance induction. It also suggests that people who pre-exposed to ATEC or DEHP should be careful not to be exposed repeatedly to the same one. So, we recommend that it should be better to use carefully ATEC as substitute plasticizer for DEHP.

Based on our previous report^4^, *in vitro* 10^−5^ M of DEHP can be converted to 3,900.6 μg/L ≒ 4.0 mg/kg *in vivo*. Although European Food Safety Authority (EFSA) and Republic of Korea permitted 50 μg/kg of DEHP per day legally (EFSA, 2005a, 2005b), it may not rule out the possible risk by long-term continuous and repeated exposure to DEHP. So, the experimental condition should be reflected a daily exposure situation to be taken by various routes such as mouth, skin, respiration and so on in our living system. Then, we used exceptional concentration for another reason that ATEC and DEHP gradually decreased by absorption, distribution, metabolism and excretion after oral administration. In our *in vivo* experiments, mice were injected with ATEC or DEHP multiplied by 10, 100 and 500 from 4.0 mg/kg DEHP for 5 days. It is possible to explain a correlation between the lowest *in vivo* dose, 4.0 mg/kg used in this study and the dose 50 μg/kg per day of human daily exposure, which is a tolerable daily intake (TDI) in EFSA and Republic of Korea^35,36^. 4, 40, 400 and 2,000 mg/kg DEHP should be about 80, 800, 8,000 and 40,000 fold to human TDI basis. In addition, DEHP of 4, 40, 400 and 2,000 mg/kg could be also converted to about 100 μg, 1, 10 and 50 mg administered for an individual mouse with 25 g, average body weight. These absolute amounts are about 2, 20, 200 and 1,000 fold higher in mouse than TDI, 50 μg/kg body weight/day in human. So, we thought this is acceptable to reflect long-term and repeated exposure because ATEC and DEHP in our experiments were injected only for 5 days.

We question why glucose tolerance induction by ATEC and DEHP was different depending on period and amount of their exposure; no changes in BGL by 4, 40, 400 mg/kg and a significant change in BGL by 2,000 mg/kg for 5 consecutive administration per day; significant changes in BGL by 4, 40, 400 mg/kg and no changes in BGL by 2,000 mg/kg for an additional single administration 7 days after the last administration of 5 consecutive administration. Data demonstrate that although 2,000 mg/kg is very high dose, no effect on BGL was observed by an additional single administration but the repeated exposure is strong to change BGL. However, an additional single administration with even lower dosages to pre-exposed individual is effective on the changes in BGL. So, it is required to study the different *in vivo* effect and to clarify further reason on BGL between the first repeated exposures with different doses.

Many tumor types consumed glucose at an extraordinarily high rate, which was called ‘Warburg effect’. Glucose provides the source for a diverse array of cellular functions. Then, tumor cells acquire the unique pattern of metabolic enzymes and regulation that non-transformed cells use as sparingly as possible^37^. DEHP also promote EMT and cancer cell metastasis^38^, which may lead to enhance colon or hepatic tumorigenesis^10–12^. Then, US EPA classifies DEHP as probable human carcinogens^13^. DEHP-induced oxidative stress may induce inflammation, the expression of protooncogenes and tumorigenesis in PPARα-null mice^11^. Tumor microenvironment (TME) supports inappropriate metabolic reprogramming that impacts the antitumor immune response and tumor progression^39^. DEHP also reduces tumor-preventing ability by the suppression of *in vivo* immune responses of macrophages^4^. So, it is possible for metabolic changes by glucose tolerance induction by ATEC to be associated with the initiation of tumor formation and the rate of tumor growth like in DEHP-exposed mice.

In conclusion, although we could not explain the different effect on BGL between different doses and ATEC did not induce genotoxicity in bacterial and cellular system, metabolic changes by glucose tolerance induction were higher in ATEC-treated group than that in DEHP-treated group. So, it should be careful to mention whether ATEC could be safer substitute plasticizer than DEHP in the face of metabolic changes. In addition, these results suggest that the companies should reduce or stop the use of DEHP or even substitutes such as ATEC and then contribute to decrease their contamination in the nature.
